# Current advances on RIPK2 and its inhibitors in pathological processes: a comprehensive review

**DOI:** 10.3389/fnmol.2025.1492807

**Published:** 2025-05-08

**Authors:** Shanshan Shen, Chen Lu, Tao Ling, Yanan Zheng

**Affiliations:** ^1^Jinhua Hospital Affiliated to Zhejiang University School of Medicine, Jinhua, China; ^2^Sir Run Run Hospital, Nanjing Medical University, Nanjing, China; ^3^Second Affiliated Hospital of Nanjing Medical University, Nanjing, China

**Keywords:** RIPK2, nod-like receptors, protein structure, RIPK2 inhibitors, ubiquitin signaling

## Abstract

Receptor-Interacting Protein Kinase 2 (RIPK2) is a critical component of the signaling pathways downstream of Nucleotide-binding oligomerization domain-like receptor (NOD-like receptor), playing a vital role in the immune response, particularly in the context of cellular transport, adaptive immunity, and tumorigenesis. Recent advances have further clarified the complex roles of RIPK2, offering insights into its structural and functional characteristics. In this review, we provide a comprehensive overview of RIPK2’s involvement in signaling, examine the development of RIPK2 inhibitors, and discuss novel strategies for targeting RIPK2 in therapeutic applications. Additionally, we highlight the dynamic interactions between RIPK2 and NOD-like receptors and explore future directions for improving RIPK2-targeted therapies.

## 1 Introduction to RIPK2

The Receptor-Interacting Protein Kinase (RIPK) family belongs to the Tyrosine Kinase-Like (TKL) superfamily of serine/threonine kinases, consisting of seven members (RIPK1-RIPK7). These kinases share homology within their kinase domains, while sequence variations in non-kinase regions confer their distinct functions. Among them, RIPK2 is particularly significant.

RIPK2’s kinase domain was identified between 1998 and 2000 through sequence alignments and was initially categorized as a serine/threonine kinase. It was known by various names—RICK, RIP2, CARDIAK, and CCK (Thome et al., 1998; McCarthy et al., 1998; Inohara et al., 1998; Nachbur et al., 2015; Medzhitov and Janeway, 2000), but today is referred to as RIPK2. The human RIPK2 protein has 540 amino acids and is primarily localized in the cytoplasm. Due to its tyrosine autophosphorylation capacity, RIPK2 was later reclassified as a dual-specificity kinase (Tigno-Aranjuez et al., 2010). Early studies showed that RIPK2 interacts with CD95 to mediate apoptosis (Inohara et al., 1998) and plays a critical role in caspase activation (Thome et al., 1998). Further research confirmed RIPK2’s critical role as a downstream signaling molecule of NOD-like receptors (Chen et al., 2009; Strober and Fuss, 2011; Philpott et al., 2014; Hall et al., 2008; Park et al., 2007). Emerging evidence also suggests RIPK2 has functions independent of NOD-like receptors signaling.

In the early characterization of RIPK2, researchers created three kinase-inactive mutants: K38M (Inohara et al., 1998), K47A (McCarthy et al., 1998), and D146N (Thome et al., 1998). Interestingly, one study reported a truncated version of RIPK2 lacking nine amino acids (NGEAICSAL) in the K38M mutant. Upon further analysis, K38M and K47A were found to involve mutations at the same lysine residue, which is crucial for ATP binding by forming a salt bridge with the αC helix (Kornev and Taylor, 2010).

## 2 Structure of RIPK2

### 2.1 Basic structure of RIPK2

#### 2.1.1 RIPK2 consists of three main domains

**Figure 1:**
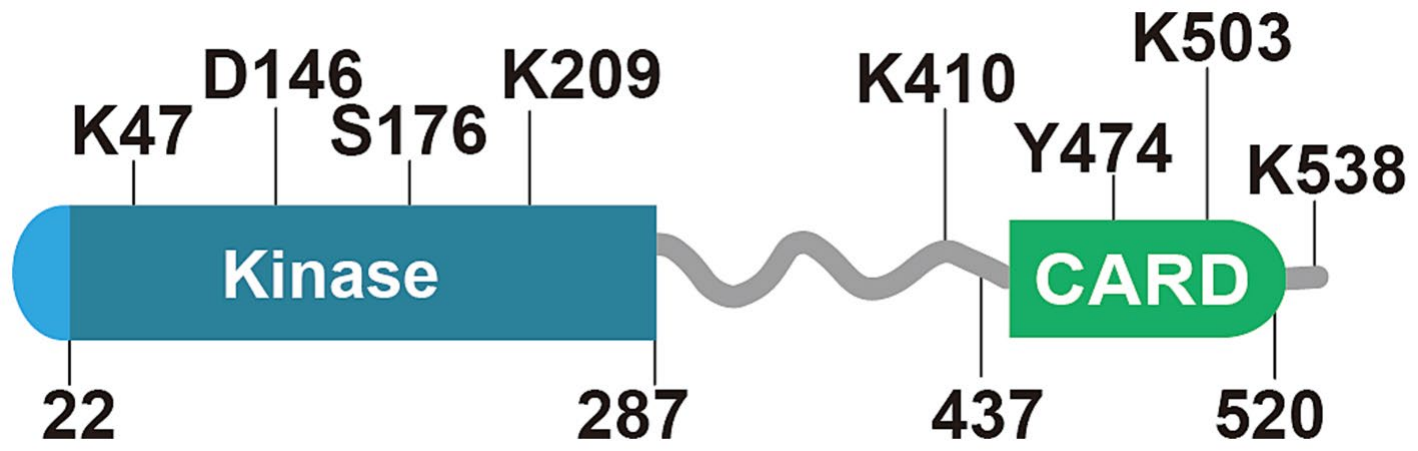
Key functional domains and regulatory sites of RIPK2. RIPK2 contains a kinase domain (aa22-287) and a CARD domain (aa437-520). Residues K47/D146 are critical for kinase activity; their mutation (K47A/D146N) abolishes function. S176 phosphorylation activates NLR signaling, while K209 ubiquitination drives NF-κB activation (blocked in K209R mutants). Dual mutation K410/538R suppresses NLR signaling. In the CARD domain, Y474 phosphorylation enables RIPosome assembly, whereas K503 ubiquitination (ZNRF4-mediated, K48-linked) promotes proteasomal degradation to regulate NOD2 tolerance. This figure summarizes essential modifications controlling NLR signaling and RIPK2 stability.

The kinase domain (KD), the intermediate domain, and the caspase activation and recruitment domain (CARD) (Chen et al., 2009; Strober and Fuss, 2011; Philpott et al., 2014; Hall et al., 2008; Park et al., 2007) (Figure 1). The interaction between RIPK2 and NOD-like receptors occurs through CARD-CARD interactions (Park et al., 2007; Girardin et al., 2001). Specifically, the CARD domains of NOD1 and NOD2 bind with the CARD of RIPK2, forming CARD-CARD complexes (Chen et al., 2009; Strober and Fuss, 2011; Philpott et al., 2014; Strober et al., 2006). NOD2’s CARD has two basic residues (R38, R86) that interact with acidic residues on RIPK2 (D461, E472, D473, E475, D492). On the other hand, NOD1’s interaction with RIPK2 involves three acidic residues on NOD1 and three basic residues on RIPK2 (R444, R483, R488) (Manon et al., 2007). Additionally, two more residues on RIPK2 (K443, Y474) have been found to be essential for its interaction with NOD1 (Mayle et al., 2014). Notably, the NOD2-RIPK2 interaction has primarily been observed in recombinant protein systems or overexpression models.

### 2.2 Three-dimensional structure of RIPK2

Since 2015, the three-dimensional structures of both the kinase domain and CARD of RIPK2 have been progressively elucidated (Lin et al., 2015; Goncharuk et al., 2018; Canning et al., 2015; He et al., 2017; Hrdinka et al., 2018; Suebsuwong et al., 2018). The kinase domain of RIPK2 adopts a canonical kinase fold, with its catalytic core residing between the N-terminal lobe and C-terminal lobe, making it a primary target for therapeutic intervention. The C-terminal CARD of RIPK2 exhibits typical characteristics of the death domain superfamily, albeit with a distinctive additional sixth helix not observed in other CARDs or death domains. The intermediate domain of RIPK2, characterized by its high flexibility, remains poorly understood in terms of its potential influence on the overall function of the protein.

**Figure 2:**
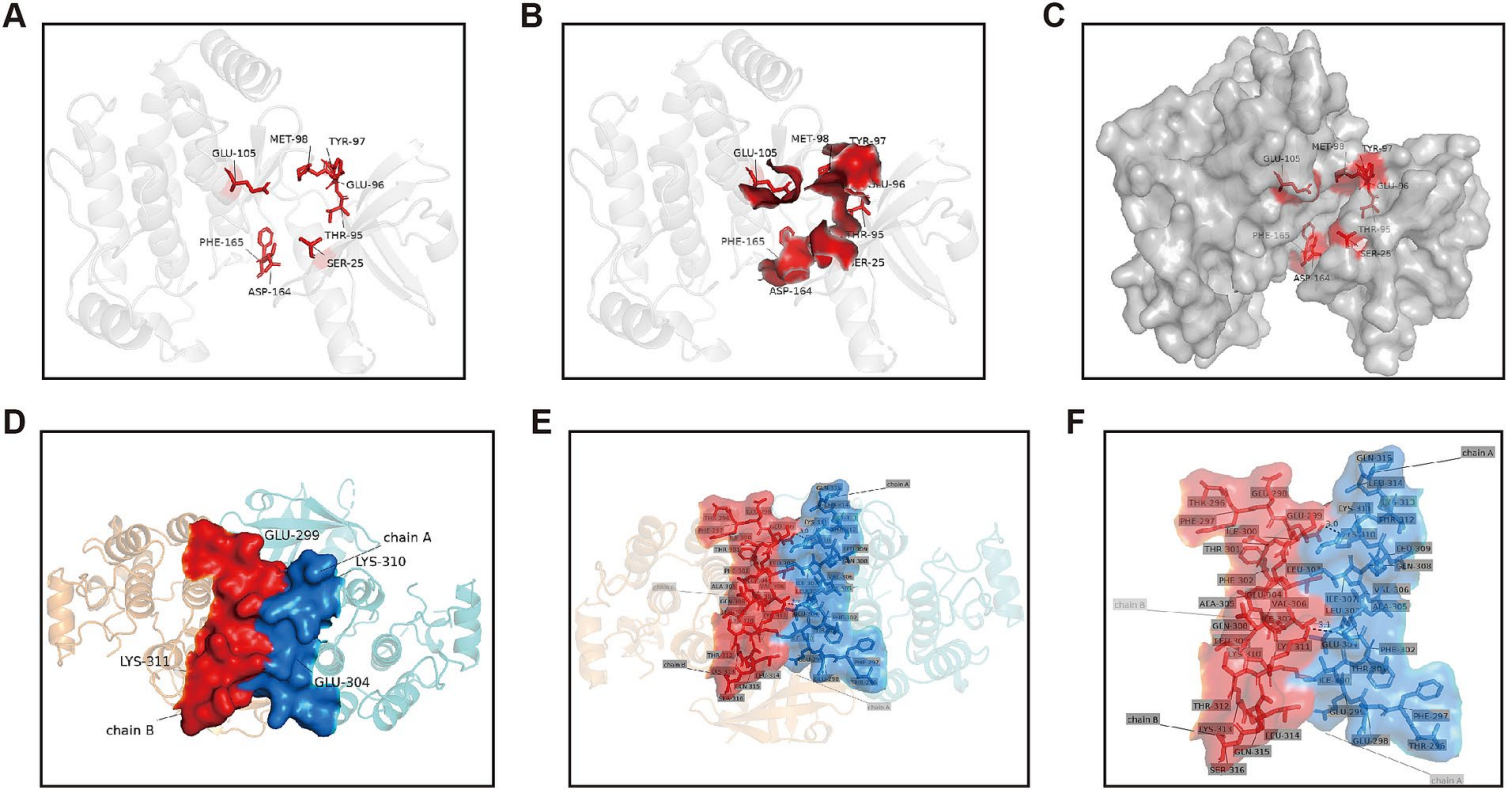
ATP-binding pocket and dimerization interface of the RIPK2 protein. **(A)** Key amino acid residues forming the ATP-binding pocket of RIPK2. **(B)** Surface representation highlighting the residues within the ATP-binding pocket. **(C)** Overall view of RIPK2 with surface rendering showing the location of the ATP-binding pocket. **(D)** Interface of the RIPK2 dimerization site. **(E)** Detailed view of the dimerization interface, illustrating key residues and hydrogen bonds. **(F)** Enlarged view of panel **(E)** showing the interaction details.

Within the structural architecture of RIPK2, two critical binding interfaces warrant particular attention. The first is the ATP-binding pocket within the kinase domain, which serves as the focal point for the design of numerous inhibitors (Figures 2A–C). The second is the *α*-helix (αJ) comprising residues E299-K310, which is pivotal for the dimerization or oligomerization of RIPK2 (Figures 2D–F).

### 2.3 RIPK2 oligomerization

This passage suggests that RIPK2 mainly operates in the form of dimers or oligomers. In its active state, RIPK2 forms a stable dimer, while in its inactive state, it exists in an equilibrium between monomers and dimers. Recent crystal structures show that dimerization is important for activating the kinase function of RIPK2 (Nachbur et al., 2015; Tigno-Aranjuez et al., 2010; Canning et al., 2015; Tigno-Aranjuez et al., 2014; Charnley et al., 2015; Haile et al., 2016). The formation of RIPK2 dimers is dependent on their respective αJ helices (Figure 2), which are stabilized by hydrophobic interactions between the side chains of Lys310 and Glu299 and the side chains of His159 and Glu157, as well as a symmetric hydrogen bond network.

In contrast to RIPK2 dimers, the formation of RIPK2 oligomers may rely more heavily on its CARD domain. During certain bacterial infections, RIPK2 oligomerizes in the cytoplasm, forming a helical structure composed of 12 RIPK2-CARD monomers, which is referred to as the “RIPosome” (Gong et al., 2018; Pellegrini et al., 2018; Kornelia et al., 2019). The formation of the RIPosome is dependent on the CARD domain of NOD1/2, which is responsible for recruiting RIPK2. The filamentous structure formed by RIPK2 aggregation is thought to act as a signaling platform downstream of NOD-like receptor. Following invasive bacterial infection, the RIPosome appears in the cytoplasm and can increase over time. Although the precise function of the RIPosome remains unclear, existing data suggest that it may serve as a platform for downregulating RIPK2 signaling (Kornelia et al., 2019). Additionally, the phosphorylation of RIPK2 at the Y474 site is essential for RIPosome formation, while the absence of phosphorylation at the S176 site promotes its formation (Kornelia et al., 2019). Interestingly, RIPK1 and RIPK3 can also form higher-order molecular complexes, known as the “RIPoptosome,” which distinguish between necroptosis and apoptosis (Kornelia et al., 2019; Feoktistova et al., 2011). However, Ellwanger et al. demonstrated in a HeLa cell model that the RIPosome is not directly associated with apoptosis (Kornelia et al., 2019).

**Figure 3:**
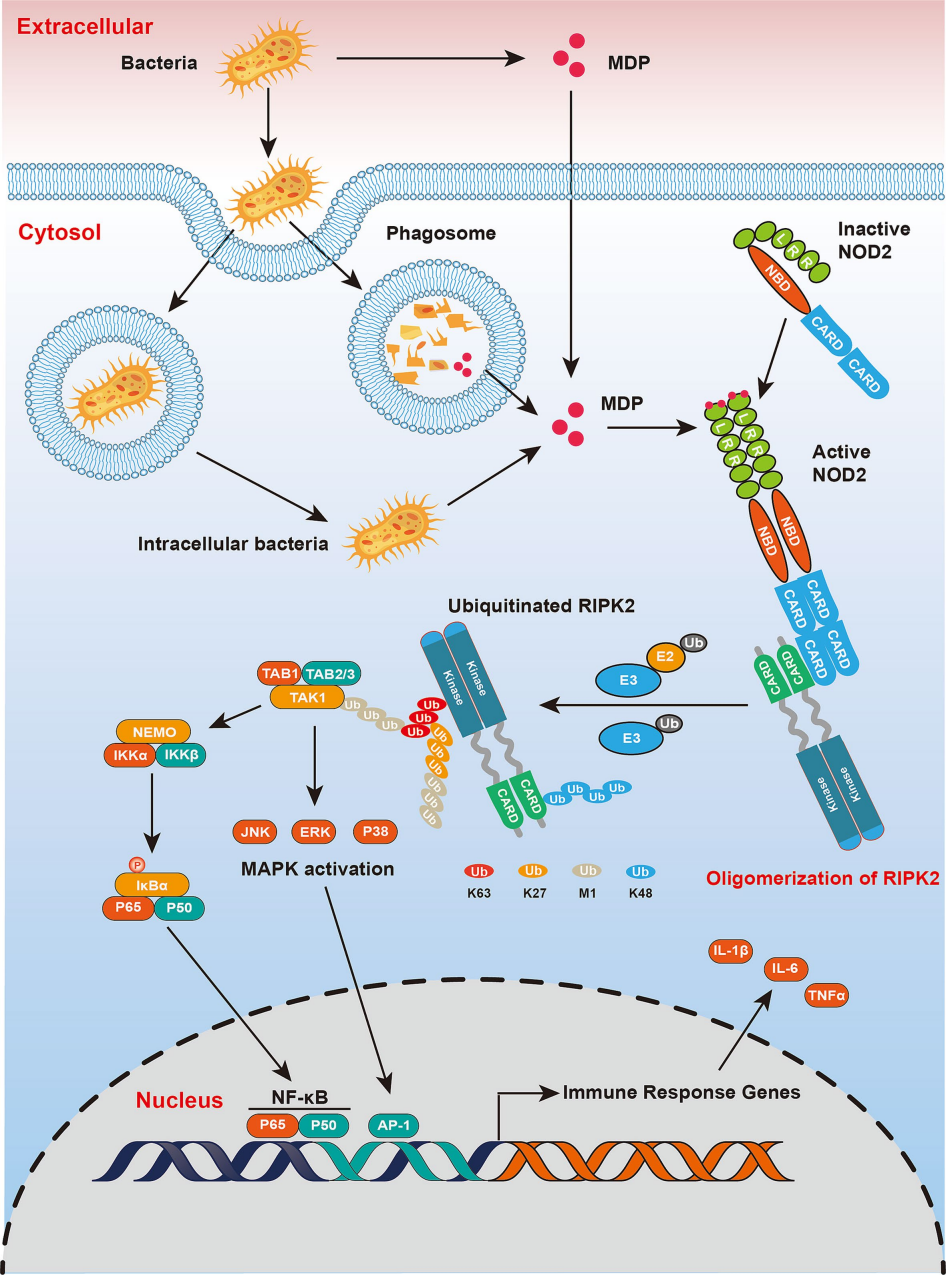
NOD2 receptor signaling pathway. The receptor NOD2, which detect bacterial components, play a critical role in inflammatory signaling by driving the ubiquitination of RIPK2. Upon ligand engagement, RIPK2 is recruited through CARD-CARD domain interactions, leading to its ubiquitination facilitated by E3 ubiquitin ligases. These ligases generate K63-, K27- and M1-linked ubiquitin chains. The chains are then recognized by kinase complexes that are dependent on ubiquitin, specifically the TAK1-TAB complex and the IKKα/*β*-NEMO complex. Activation of TAK1 triggers MAP kinase signaling cascades, while IKK phosphorylates the NF-κB inhibitor IκB, marking it for ubiquitination (via K48-Ub chains) and subsequent degradation by the proteasome. This process culminates in the nuclear translocation of the transcription factor NF-κB, which, together with AP-1, drives the expression of genes that orchestrate inflammatory and immune responses, including those encoding pro-inflammatory cytokines and chemokines.

Based on the above information, primarily referring to the fact that RIPK2 typically exists as a dimer in its active state and forms a dodecamer through CARD-CARD interactions *in vitro*, we propose the following model: after bacterial invasion, the CARD domain of NOD1/2 recruits RIPK2, leading to the aggregation of RIPK2 into a dodecamer composed of dimers as subunits. This process is transient, and the oligomer acts as a signaling platform by recruiting ubiquitin ligases and downstream kinases to promote cytokine transcription. Subsequently, the oligomer undergoes degradation via K48-mediated ubiquitin-proteasome pathways or loses activity through caspase-1-mediated cleavage of the kinase domain, resulting in intracellular RIPK2 protein depletion and tolerance in the NOD-like receptor signaling pathway (Figure 3).

## 3 The function of RIPK2

### 3.1 RIPK2 as a key downstream signaling molecule of NOD-like receptors signaling pathway

RIPK2 is a key signaling molecule downstream of NOD-like receptor. It interacts with NOD1 and NOD2 through CARD-CARD interactions (Chen et al., 2009; Philpott et al., 2014; Strober et al., 2006; Strober et al., 2014; Strober and Watanabe, 2011). Upon NOD activation, RIPK2 undergoes various types of ubiquitination at multiple sites, which is crucial for the activation of the NF-κB and mitogen-activated protein kinase (MAPK) pathways. Overexpression of RIPK2 in models such as macrophages and *Mycobacterium tuberculosis* infection revealed that NOD2 signaling involves K63-linked polyubiquitination of RIPK2 (Yang et al., 2007), critical for NOD2 pathway activation (Hasegawa et al., 2008).

#### 3.1.1 Ubiquitination as a key function of RIPK2 in the NOD-like receptor signaling pathway

Ubiquitination of RIPK2 plays a critical role in the activation of downstream signaling proteins within the NOD1/2 pathway. RIPK2 recruits TAK1 through the LUBAC complex (Kanayama et al., 2004), which subsequently brings in TAK1-binding proteins TAB2 and TAB3, initiating the MAPK signaling cascade. TAK1 also triggers IKK activation, leading to the degradation of p-IκBα and activation of NF-κB (Hasegawa et al., 2008; Inohara et al., 2000; Yang et al., 2007) (Figure 3).

**Figure 4:**
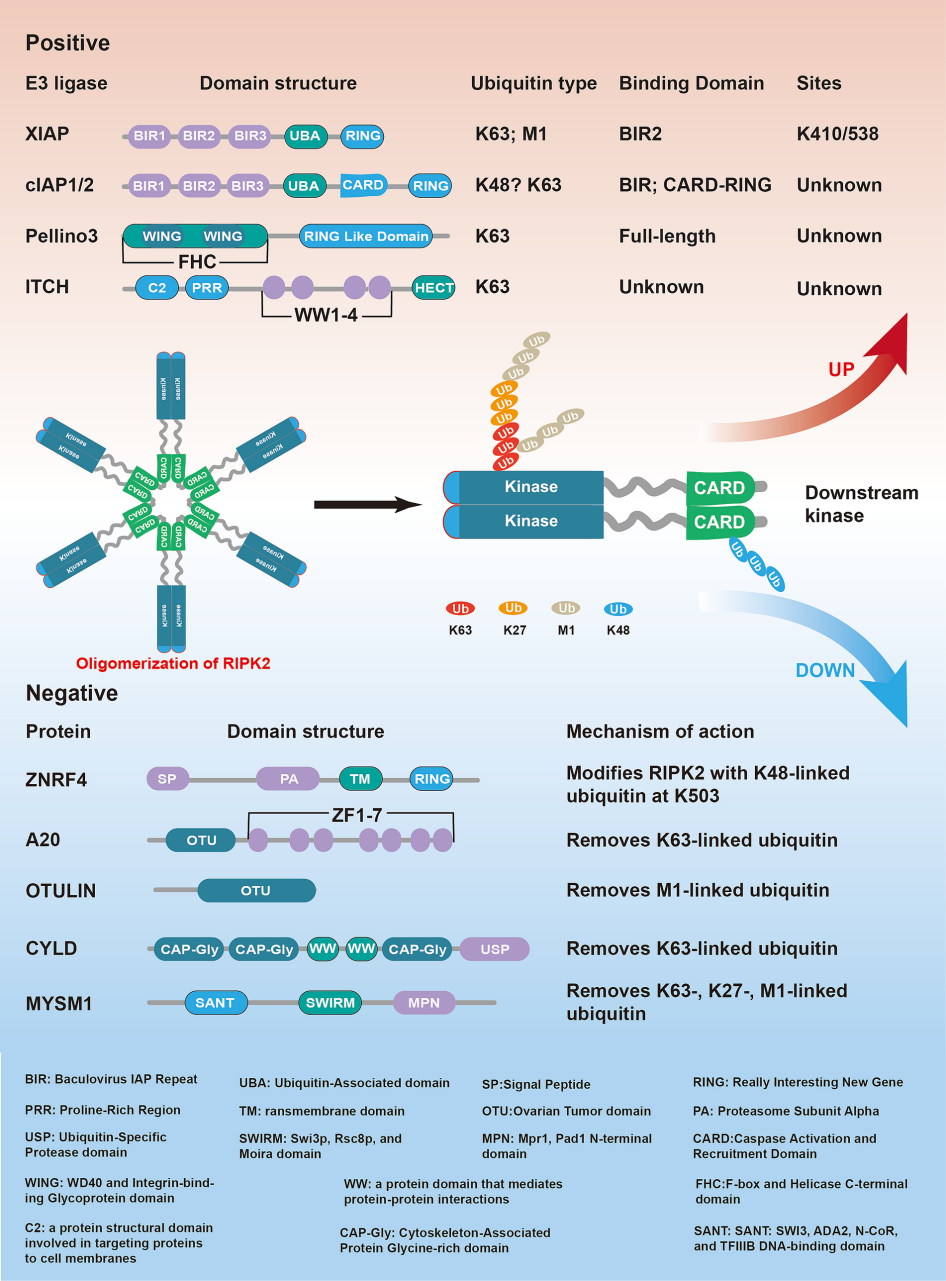
The ubiquitination of RIPK2 is essential for regulating NF-κB and MAPK activation downstream of NOD1 and NOD2. Upon ligand binding, RIPK2 is rapidly ubiquitinated with K63-, K27- and M1-linked polyubiquitin chains. The E3 ligase XIAP are critical for triggering downstream signaling. Other E3 ligases, such as c-IAP1, c-IAP2, pellino3 and ITCH also contribute to the ubiquitination of RIPK2. Conversely, negative regulation of NODs signaling is mediated by both deubiquitinases and ubiquitin ligases that promote RIPK2 degradation. Deubiquitinases like A20, OTULIN, CYLD, and MYSM1 remove ubiquitin chains from RIPK2, while ZNRF4 negatively regulates NOD signaling by promoting RIPK2 degradation through K48-linked ubiquitination.

During NOD-like receptor signaling, RIPK2 ubiquitination involves various types, including K63, M1, K48, and K27 (Damgaard et al., 2012; Panda and Gekara, 2018). The Cellular Inhibitor of Apoptosis (IAP) family comprising cIAP1, cIAP2 and XIAP, regulates NOD-like receptor signaling by mediating the ubiquitination of RIPK2 (Bertrand et al., 2009; Krieg et al., 2009). cIAP1, cIAP2, and XIAP contain a ubiquitin-associated domain (UBA) capable of binding ubiquitin chains, and a RING domain with E3 ubiquitin ligase activity (Silke and Vucic, 2014) (Figure 4).

The involvement of cIAPs in NOD-like receptor signaling was first discovered in 1998, when overexpressed cIAPs were co-immunoprecipitated with RIPK2 in HEK 293 T cells (Thome et al., 1998). Subsequent studies confirmed that cIAP1 and cIAP2-deficient mice had significantly reduced cytokine responses to MDP injection. These proteins can also ubiquitinate RIPK2 independent of its CARD domain (Bertrand et al., 2009). However, the exact role of cIAPs in NOD signaling remains debated. Some studies have reported that knocking out cIAP1/2 does not significantly affect NOD2 receptor signaling (Damgaard et al., 2012; Stafford et al., 2018). Although cIAP1/2-deficient mice show an impaired response to MDP, signaling transduction *in vitro* remains intact (Stafford et al., 2018). Recent evidence suggests that while cIAP1/2 may not play a critical role in this pathway, XIAP is essential for RIPK2 ubiquitination and signaling, particularly through K63 and M1-linked ubiquitin chains (Damgaard et al., 2012; Krieg et al., 2009). In cells and mice lacking XIAP, RIPK2 ubiquitination is significantly reduced, impairing NOD signaling. SPR experiments have shown a direct interaction between XIAP’s BIR2 domain and RIPK2’s kinase domain (Goncharov et al., 2018). Targeting the XIAP BIR2 domain with antagonists disrupts this interaction, reducing RIPK2 ubiquitination and impairing MAPK and NF-κB signaling (Hrdinka et al., 2018; Goncharov et al., 2018).

K63-linked ubiquitination of RIPK2 is crucial for LUBAC recruitment (Damgaard et al., 2012). LUBAC is the only known protein complex capable of adding linear ubiquitin chains to substrates (Fiil et al., 2013; Tokunaga, 2013). It is currently hypothesized that linear ubiquitin chains are added as branches onto K63-linked ubiquitin chains (Panda and Gekara, 2018). Additionally, XIAP can prevent the formation of RIPosomes, as XIAP knockdown leads to the spontaneous formation of RIPosomes (Kornelia et al., 2019; Goncharov et al., 2018; Lesage et al., 2002). Interestingly, ubiquitination also plays a role in the activation of RIPK1 and RIPK3, with XIAP deficiency leading to the spontaneous formation of the RIPoptosome (Tenev et al., 2011; Yabal et al., 2014). Other E3 ligases that have been reported to mediate RIPK2 ubiquitination and promote NOD-like receptor signaling include TNF receptor-associated factor 6 (TRAF6), TNF receptor-associated factor 2 (TRAF2), and TNF receptor-associated factor 5 (TRAF5). These proteins contain RING domains and are associated with the NOD-like receptor signaling pathway (Xie, 2013). Unfortunately, there is currently no evidence to suggest that TRAF2 and TRAF5 directly ubiquitinate RIPK2 in the NOD-like receptor signaling, and the conclusion that TRAF6 promotes K63-linked ubiquitination of RIPK2 has been challenged in subsequent studies (Yang et al., 2007; Damgaard et al., 2012; Bertrand et al., 2009; Tao et al., 2009). Although siRNA knockdown of TRAF6 in HEK 293 T cells reduced RIPK2 ubiquitination and NF-κB activation after NOD2 stimulation (Yang et al., 2007), TRAF6-deficient mouse embryonic fibroblasts still activate NF-κB and MAPK in response to NOD1 agonists (Hasegawa et al., 2008).

The E3 ubiquitin-protein ligase Pellino homolog 3 (Pellino3) facilitates K63-linked polyubiquitination of RIPK2. Bone marrow-derived macrophages (BMDMs) from Pellino3-deficient mice showed reduced NF-κB and MAPK activation and lower cytokine production after MDP stimulation (Yang et al., 2013). Notably, Pellino3 expression was reduced in the colons of Crohn’s disease patients, supporting its role as an important mediator of NOD2 receptor signaling in the intestine. *In vitro* experiments also identified the E3 ubiquitin-protein ligase Itchy homolog (ITCH) as another direct ligase for RIPK2 (Tao et al., 2009). BMDMs from ITCH knockout mice failed to ubiquitinate RIPK2, leading to reduced NF-κB and MAPK activation and decreased NF-κB target gene expression after MDP stimulation.

While ubiquitination plays a critical role in the activation of NOD-like receptor signaling, it is equally important in regulating the downregulation of RIPK2 activity. This balance between activation and inhibition ensures proper immune responses and prevents excessive inflammation.

#### 3.1.2 Negative regulation of NOD-like receptor signaling pathway via RIPK2 ubiquitination

The regulation of NOD-like receptor signaling via RIPK2 ubiquitination has been a key research focus. Two main mechanisms are involved: K48-linked polyubiquitination leading to RIPK2 degradation, and deubiquitination by specific enzymes. A genome-wide RNAi screen in HEK 293 T cells identified zinc/RING finger protein 4 (ZNRF4) as a negative regulator of NOD2-dependent NF-κB activation. ZNRF4 promotes RIPK2 degradation via K48-linked ubiquitination. Macrophages with ZNRF4 knockdown produce higher levels of pro-inflammatory cytokines in response to MDP and show reduced tolerance to secondary exposure to MDP and *Listeria monocytogenes* (Bist et al., 2017). These findings suggest ZNRF4 may be part of a negative feedback loop that limits prolonged NOD2 receptor signaling. RIPK2 degradation could be one of the mechanisms maintaining NOD2 receptor signaling tolerance (Figure 4).

Deubiquitinating enzymes (DUBs) regulate NOD2 signaling by removing ubiquitin chains from RIPK2. Zinc finger protein A20 was the first DUB identified to negatively regulate NOD2 signaling by removing non-K48-linked ubiquitin chains from RIPK2 (Hitotsumatsu et al., 2008). Ubiquitin thiolesterase OTULIN removes M1-linked ubiquitination from RIPK2, counteracting LUBAC and limiting NOD2-induced NF-κB and MAPK signaling (Fiil et al., 2013). Ubiquitin carboxyl-terminal hydrolase CYLD targets M1- and K63-linked ubiquitin chains, thereby restricting NOD2 signaling (Hrdinka et al., 2016). Deubiquitinase MYSM1 specifically removes K27-, K63-, and M1-linked ubiquitin chains, reducing NOD2 signaling. In MYSM1-deficient mice, increased neutrophil recruitment after MDP injection suggests MYSM1 as a negative regulator of NOD signaling (Panda and Gekara, 2018). Thus, the fine-tuning of RIPK2 ubiquitination, both in promoting degradation and removing activating ubiquitin chains, ensures that NOD signaling does not persist unchecked, maintaining immune tolerance and preventing pathological inflammation (Figure 4).

During the investigation of RIPK2 ubiquitination, several key lysine residues were identified. Ubiquitination at K209 is crucial for NOD2 receptor signaling, as the RIPK2 K209R mutant failed to activate NF-κB (Hasegawa et al., 2008). In addition to K209, XIAP-dependent ubiquitination sites were identified at K410 and K538. Mutation of these sites (K410/538R) led to reduced NF-κB activation and cytokine production. Furthermore, ZNRF4 promotes K48-linked polyubiquitination at K503, facilitating RIPK2 degradation and contributing to NOD2 pathway tolerance (Bist et al., 2017).

Beyond the regulation of RIPK2 by ubiquitination, its intrinsic kinase activity has also been the subject of much debate, as researchers aim to understand whether it plays a direct role in NOD-like receptor signaling.

#### 3.1.3 The role of RIPK2 kinase activity in NOD-like receptor signaling pathway

The role of RIPK2 kinase activity in NOD-like receptor signaling has been debated for over 26 years. Only recently has a consensus begun to form. To clarify, four key questions must be addressed:

1) Does a kinase-dead RIPK2 mutant affect NOD-like receptor signaling? In 2018, Goncharov et al. addressed this question (Goncharov et al., 2018). They introduced either WT RIPK2 or D146N RIPK2 (a kinase-dead mutant) into HEK-Blue-hNOD2 RIPK2^−/−^ cells and found no difference in NOD2 signaling activation, demonstrating that RIPK2 kinase activity is not required for NOD2 signaling. However, BMDMs from kinase-dead (K47A) knock-in mice exhibited defects in NOD-like receptor signaling (Goncharov et al., 2018), likely due to low expression of the mutant. This suggests that RIPK2 kinase activity may be important for protein stability rather than for NOD-like receptor signaling itself. Additionally, the K47A mutant disrupts RIPK2 dimerization (Pellegrini et al., 2017), likely impairing the overall signaling structure.
2) Does kinase-inactive RIPK2 affect its ubiquitination? Goncharov et al. demonstrated that the D146N RIPK2 mutation did not affect polyubiquitination of RIPK2 during NOD2 signaling, further supporting the idea that the kinase domain of RIPK2 functions merely as a scaffold in NOD-like receptor signaling (Goncharov et al., 2018). These findings further support the notion that RIPK2’s kinase domain serves a scaffolding function, facilitating its ubiquitination without directly driving signaling.
3) How do RIPK2 kinase inhibitors impact NOD-like receptor signaling and ubiquitination? Some RIPK2 kinase inhibitors block NOD-like receptor signaling and RIPK2 polyubiquitination (Nachbur et al., 2015; Goncharov et al., 2018). However, studies by Goncharov et al. and Hrdinka et al. suggest this inhibition occurs by disrupting RIPK2’s interaction with the XIAP BIR2 domain, rather than directly inhibiting its kinase activity. The observed inhibition is likely due to disruption of RIPK2-XIAP interactions, rather than direct kinase inhibition.
4) Does RIPK2 autophosphorylation affect its ubiquitination and NOD-like receptor signaling? Upon CARD-CARD interaction-based activation, RIPK2 undergoes phosphorylation at S176 in its kinase domain and Y474 within its CARD domain (Tigno-Aranjuez et al., 2010; Goncharov et al., 2018). Mutations at these sites impair RIPK2’s ability to trigger downstream signaling, particularly in overexpression systems. In HeLa cells, both wild-type RIPK2 and the S176A mutant induced comparable cytokine levels following infection by *Shigella flexneri*, whereas the S176E mutant exhibited reduced cytokine levels. The Y474F mutation led to the complete inhibition of cytokine production (Kornelia et al., 2019). Although Nembrini et al. proposed that phosphorylation at these sites affects signaling (Pellegrini et al., 2017), studies using the D146N kinase-dead mutant found no impact on NOD2 signaling, suggesting these phosphorylation sites do not influence signal transduction (Goncharov et al., 2018). Cryo-EM studies provided further insights, demonstrating the critical importance of Y474 in RIPK2 signaling. Y474 resides at a vital interface within the CARD domain, facilitating interactions required for oligomerization and NF-κB activation. Substituting Y474 with phenylalanine disrupts RIPK2 activity, highlighting its role in signaling (Gong et al., 2018; Pellegrini et al., 2018).

In summary, while RIPK2 kinase activity appears to contribute to protein stability and oligomerization, its role in directly driving NOD-like receptor signaling remains limited, with its primary function being scaffolding for ubiquitination and downstream activation.

### 3.2 RIPK2 and innate immunity

Members of the RIPK family play a broad role in activating innate immune responses, particularly in the activation of the transcription factor NF-κB. RIPK2 was first identified as a positive regulator of NF-κB (Thome et al., 1998). Several studies have demonstrated increased RIPK2 mRNA expression following bacterial infections, including *Legionella pneumophila* (Frutuoso et al., 2010), *Mycobacterium tuberculosis* (Divangahi et al., 2008), *Listeria monocytogenes* (Kobayashi et al., 2002), *Salmonella enterica* (Geddes et al., 2010), and *Chlamydia pneumoniae* (Shimada et al., 2009; Shehat et al., 2019). In RIPK2-deficient mice, NF-κB activation is impaired, leading to decreased IL-6 and TNF-*α* expression, reduced neutrophil infiltration, and a diminished ability to resist intracellular pathogens (Kobayashi et al., 2002; Chin et al., 2002).

### 3.3 RIPK2 and adaptive immunity

RIPK2 plays a critical role in adaptive immune responses (Lupfer et al., 2013; Meylan and Tschopp, 2005; Van Gorp and Lamkanfi, 2019). Studies show that RIPK2-deficient mice exhibit increased IL-18 secretion and heightened inflammatory responses following influenza A virus infection, and secondary bacterial infections trigger RIPK2 expression, potentially driving uncontrolled immune responses (Chin et al., 2002; Lupfer et al., 2013).

The role of RIPK2 in adaptive immunity remains debated. Early studies indicated RIPK2 may promote Th1 differentiation and regulate Th1 and NK cell responses to IL-12 and IL-18, enhancing IFN-*γ* production (Kornelia et al., 2019; Chin et al., 2002). Subsequent studies, however, indicated that this effect might be indirect, mediated by RIPK2’s influence on the NOD-like receptor signaling pathway, rather than a direct action on Th1 cell differentiation or graft rejection (Fairhead et al., 2008). Recent research highlights RIPK2’s regulatory role in Th17 differentiation, suggesting that T cell-intrinsic RIPK2 is key for maintaining Th17 homeostasis and preventing over-differentiation (Shimada et al., 2018). While promising, further research is needed to fully understand RIPK2’s complex role in adaptive immune regulation.

In addition to its regulatory functions in both innate and adaptive immunity, RIPK2 also plays a key role in determining cell fate, influencing processes such as autophagy, cell death, and proliferation.

### 3.4 The role of RIPK2 in cell fate

RIPK2 is pivotal in controlling processes like autophagy, cell death, and proliferation. Evidence from overexpression systems suggests that during bacterial infections, the autophagy-related protein ATG16L1 might interact with RIPK2 (Cooney et al., 2010; Travassos et al., 2010), although this interaction has not been confirmed in studies of IAV infections (Lupfer et al., 2013). Mutations in ATG16L1, linked to Crohn’s disease, interfere with the interaction between NOD2 and RIPK2, resulting in the suppression of RIPK2 signaling and heightened inflammatory responses (Sorbara et al., 2013). Further research is essential to understand RIPK2’s precise mechanisms in autophagy regulation.

RIPK2 is also capable of interacting with proteins in the death receptor family, such as FADD-like IL-1β-converting enzyme inhibitory protein c-FLIP, cIAP1, cIAP2, and members of the TNFR-associated factor family, which suggests RIPK2’s involvement in regulating cell death (Thome et al., 1998). Although early studies linked the NOD-like receptor signaling pathway with caspase activation and apoptosis, more recent studies using advanced experimental models suggest that RIPK2 may regulate cell death independently of NOD-like receptor signaling. SILAC-based quantitative mass spectrometry analysis in HeLa S3 cells identified multiple specific phosphorylation sites on RIPK2 (e.g., S168, S176, S178, S345, S348, S363, Y474, T482, Y520, S527, S529, S531, and S539), which regulate the S and M phases of mitosis (Daub et al., 2008). Other studies have observed phosphorylation of the S531 site during the G1 and M phases of HeLa cells (Dephoure et al., 2008), and further research has identified additional phosphorylation sites related to the cell cycle (Olsen et al., 2010; Sharma et al., 2014). Overall, the role of RIPK2 in cell fate, from death to proliferation, requires further investigation to fully understand its mechanisms.

In conclusion, RIPK2 plays a pivotal role in immune signaling, particularly in mediating responses through NOD-like receptor pathways and regulating NF-κB activation. Its involvement in processes such as ubiquitination, kinase activity, and interactions with other CARD domain proteins highlights its multifaceted function in immune regulation and cellular homeostasis. These insights provide a solid foundation for exploring RIPK2’s broader implications, particularly in the context of human diseases. As we move forward, understanding how dysregulation of RIPK2 contributes to pathological conditions will be key to unlocking its potential in therapeutic applications.

## 4 The association of RIPK2 with diseases

### 4.1 RIPK2 and Crohn’s disease

RIPK2 plays a pivotal role in NOD activation signaling. Among the diseases linked to NOD2 receptor signaling, inflammatory bowel disease, particularly Crohn’s disease, is the most common (Philpott et al., 2014; Caruso et al., 2014). NOD2 and RIPK2, two critical components of the NOD2 signaling pathway, are abundantly expressed in intestinal epithelial and immune cells in the gut. NOD2 is essential for maintaining intestinal homeostasis, as it directly influences the growth and survival of colonic epithelial cells. However, NOD2-deficient mice do not develop spontaneous intestinal inflammation, and the myeloid and lymphoid cells in their gut remain unaffected under normal conditions (Kobayashi et al., 2005). These mice, however, show impaired bacterial clearance after oral or intragastric administration (Kim et al., 2011). Negroni et al. highlighted increased RIPK2 activation in pediatric Crohn’s disease (Negroni et al., 2009). Interestingly, RIPK2 may also contribute to NOD2-independent intestinal inflammation. Studies by Watanabe et al., using TNBS-induced colitis and DSS colitis models, showed that intestinal RIPK2 downregulation via siRNA protected against experimental colitis (Watanabe et al., 2019). Intrarectal injection of RIPK2-siRNA reduced the production of pro-inflammatory cytokines in the colon. Notably, TNBS or DSS-induced colitis was not affected by the loss of NOD1/2. Furthermore, patients with Crohn’s disease or ulcerative colitis exhibited elevated RIPK2 expression, while NOD2 expression remained unchanged in these conditions. NOD1 expression showed only a marginal increase in ulcerative colitis. These findings suggest that RIPK2 is a critical signaling molecule in chronic inflammatory bowel diseases in humans and experimental colitis in mice.

### 4.2 RIPK2 and neurological diseases

Recently, as the NOD1/2-RIPK2 signaling pathway’s neural role is revealed, RIPK2’s key part in neurological diseases enhances its translational value.

#### 4.2.1 Multiple sclerosis (MS)

Peptidoglycan (PGN), a well-established activator of NOD1/2 signaling, has been detected in systemic circulation of healthy individuals and within demyelinating lesions of multiple sclerosis (MS) patients, as well as in phagocytes of MS animal models (Schrijver et al., 2001; Visser et al., 2006; Branton et al., 2016). Evidence suggests that macrophages, dendritic cells, and neutrophils mediate PGN translocation from mucosal interfaces to the CNS (Laman et al., 2020), potentially underpinning the NOD1/2-RIPK2 signaling pathway’s neuroimmune regulatory role. cDNA microarray analysis of MS patient PBMCs showed increased *RIPK2* expression (Satoh et al., 2005). In EAE models, PGN activated CNS dendritic cells through NOD1/2-RIPK2 signaling, promoting Th17 differentiation and aggravating demyelination/neuroinflammation, while RIPK2 inhibitor WEHI-345 suppressed disease progression (Nachbur et al., 2015).

#### 4.2.2 Parkinson’s disease (PD)

Pathological *α*-Syn activates RIPK2 via NOD2 binding, driving microglial TNF-α/IL-1β release and A1 astrocyte activation, which collectively exacerbate dopaminergic neuron degeneration. *NOD2/RIPK2* knockout reduces neuroinflammation and neuronal loss, confirming RIPK2’s central role in PD pathogenesis (Seo et al., 2024). *LRRK2* (a PD-associated gene) deficiency suppresses RIPK2 phosphorylation, thereby inhibiting macrophage inflammatory responses, suggesting this interaction may represent a key mechanism through which LRRK2 exerts its pathogenic effects in PD (Yan and Liu, 2016).

#### 4.2.3 Intracerebral hemorrhage (ICH)

Inflammation following intracerebral hemorrhage (ICH) frequently contributes to secondary brain injury. In a collagenase-induced ICH mouse model, knockout of *Nod1* or *Ripk2* markedly suppresses microglial-driven neuroinflammation. The NOD1/RIPK2 signaling axis amplifies IL-1β/TNF-α production via a self-reinforcing feedback loop, ultimately driving neuronal death and cerebral edema progression (Wang et al., 2020; Larochelle et al., 2023).

In future, RIPK2 emerges as a promising therapeutic target for neuroinflammatory disorders. In multiple sclerosis models, combined NOD2/TLR7 activation boosts type I interferon signaling to dampen inflammation (Dubik et al., 2024). Stroke-induced RIPK2 upregulation and its potential brain-gut axis interactions suggest that RIPK2 inhibition may alleviate acute neural injury by modulating microglial responses (Larochelle et al., 2023). While its roles in cerebral ischemia–reperfusion and traumatic brain injury remain unclear, further research is needed.

### 4.3 RIPK2 and cancer

RIPK2 also plays a significant role in various cancers and could act as a prognostic marker. In inflammatory breast cancer (IBC), RIPK2 hyperactivation has been documented, with elevated RIPK2 and NF-κB levels observed in IBC patients even before chemotherapy. Surprisingly, chemotherapy increased RIPK2 activity, further exacerbating the molecular inflammatory response. The exact mechanism behind this hyperactivation remains unclear, but some studies suggest that HER2 and active RIPK2 in IBC are positively correlated due to the downregulation of Erbin, removing its inhibitory effect on NOD2/RIPK2 signaling. NF-κB activation has been linked to HER2 status in breast cancer. Moreover, HER2 mRNA expression in IBC patients has been found to correlate positively with RIPK2 activity (Kalkoff et al., 2004). Erbin, which interacts with Erbb2, is downregulated in HER2-overexpressing breast cancer cells (Liu et al., 2013) and it can form a complex with NOD2 to inhibit RIPK2 activity (Kufer et al., 2006). Another hypothesis involves the tumor suppressor RASSF1A, which, when hypermethylated, results in reduced expression, weakening the inhibition of NOD2/RIPK2 signaling in IBC. The loss of RASSF1A is considered a potential risk factor for IBC, with active RIPK2 possibly playing a role in the cellular response and promoting tumor progression (Volodko et al., 2016). The loss of RASSF1A is seen as a potential risk factor in IBC, with RIPK2 playing a regulatory role in tumor progression, Further studies show RIPK2’s involvement in metastasis and tumor growth, suggesting it could be a valuable prognostic marker and therapeutic target in IBC (Zare et al., 2018).

RIPK2 gene polymorphisms have also been linked to gastric cancer susceptibility (Ota et al., 2018), and increased RIPK2 expression has been associated with poor prognosis in diffuse large B-cell lymphoma (Wang et al., 2018), RIPK2 also enhances the survival of triple-negative breast cancer cells, the most aggressive subtype of breast cancer (Jaafar et al., 2018). Elevated RIPK2 expression in breast tumors correlates with poor prognosis and a higher risk of recurrence, while RIPK2 knockdown inhibits NF-κB signaling, reduces anti-apoptotic proteins, and increases drug sensitivity. Additionally, RIPK2 promotes the migration and invasion of triple-negative breast cancer cells via NF-κB and c-Jun N-terminal kinase pathways (Singel et al., 2014).

In prostate cancer, RIPK2 is highly expressed in metastatic cases and has been linked to disease progression and poor prognosis. RIPK2 knockout reduces prostate cancer invasion and metastasis significantly. RIPK2 promotes metastasis by activating MKK7 and stabilizing c-Myc, making its inhibition a potential therapeutic strategy for cancer prevention (Yan et al., 2022). Recent studies identified RIPK2 as a key driver in immune evasion in pancreatic cancer, mediating NBR1-driven MHC-I degradation, which limits antigen presentation and T cell function. Overexpressed RIPK2 in pancreatic ductal adenocarcinoma (PDAC) is associated with poor prognosis and an immunosuppressive microenvironment. Inhibiting RIPK2 enhances anti-PD-1 therapy efficacy, suggesting a combination therapy approach targeting RIPK2 and PD-1 for improved outcomes (Sang et al., 2024).

### 4.4 RIPK2 and other diseases

Beyond its role in inflammatory bowel disease and cancer, RIPK2 has also been implicated in a range of other pathological conditions. RIPK2 polymorphisms have been linked to systemic lupus erythematosus in Chinese patients and to asthma severity in the Japanese population, although the functional relevance remains to be clarified (Li et al., 2011; Nakashima et al., 2006).

Additionally, studies have shown that RIPK2-deficient mice exhibit better survival rates, improved cardiac function, and reduced cardiac hypertrophy following pressure overload. Further research indicated that RIPK2 can interact with MAVS in cardiomyocytes via CARD-CARD domain interactions, promoting NF-κB signaling, which leads to inflammation and myocardial hypertrophy (Lin et al., 2020). This suggests that RIPK2 may play an important role in cardiovascular diseases.

Collectively, these findings suggest that RIPK2 is a versatile regulator of multiple signaling pathways, influencing diverse pathological processes from immune responses to cancer progression and cardiovascular diseases. This highlights its potential as a therapeutic target in a variety of diseases.

## 5 Structural analysis of RIPK2 and targeted drug development

Given RIPK2’s involvement in various diseases, particularly those driven by dysregulated NOD-like receptor signaling, it presents a promising therapeutic target. As a result, numerous research efforts have focused on developing drugs that specifically target RIPK2.

### 5.1 Type I kinase inhibitors and their derivatives

**Figure 5:**
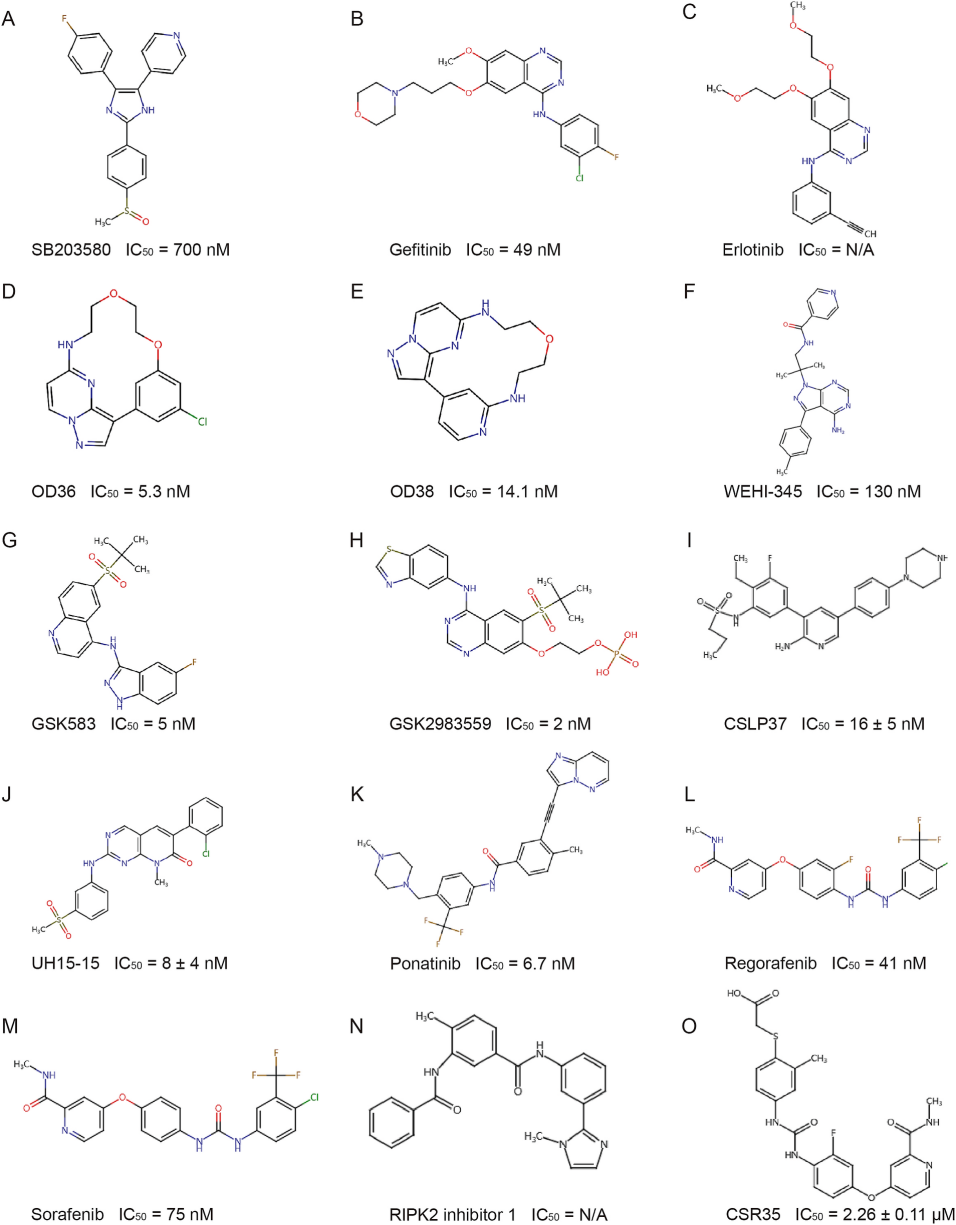
Structure of representative RIPK2 inhibitors. IC50: Half maximal inhibitory concentration. N/A: Data not available from experimental results.

Early studies indicated that RIPK2’s kinase activity is essential for NOD-like receptor signaling, prompting efforts to develop kinase inhibitors. Type I kinase inhibitors and their derivatives bind competitively to the ATP pocket, blocking RIPK2 activity. The similarity between the ATP binding sites of P38 and RIPK2 led to the use of the P38 inhibitor SB203580 (Figure 5A), which inhibited RIPK2 *in vitro* and showed efficacy in a Crohn’s disease mouse model (Argast et al., 2005; Hollenbach et al., 2005). Gefitinib and erlotinib (Figures 5B,C) also inhibited RIPK2 and reduced disease in Crohn’s-like ileitis models (Tigno-Aranjuez et al., 2010; Tigno-Aranjuez et al., 2014) though their in vitro effects on NOD2 signaling were suboptimal (Goncharov et al., 2018). Macrocyclic derivatives such as pyrazolo[1,5-a]pyrimidine (OD36) and imidazo[1,2-b]pyridazine (OD38) (Figures 5D,E) were effective at inhibiting RIPK2 autophosphorylation and downstream NF-κB/MAPK signaling (Tigno-Aranjuez et al., 2014). Furthermore, screening a compound patent library containing 120 kinase inhibitors led to the identification of WEHI-345 (Nachbur et al., 2015) (Figure 5F). Although WEHI-345 only delayed NF-κB activation and had limited inhibitory effects on NOD2 signaling in cellular models (Goncharov et al., 2018), it was beneficial in preventing nearly 50% of multiple sclerosis (MS) in a mouse model when targeting RIPK2 (Nachbur et al., 2015).

Due to the conserved ATP binding pocket, type I kinase inhibitors often lack specificity. To improve selectivity, while retaining the ATP-competitive characteristic, researchers have developed compounds that bind to structures adjacent to the ATP pocket of RIPK2 to increase selectivity. By utilizing the large hydrophobic pocket near the ATP site, GSK583 (Figure 5G) was discovered through structure–activity relationship-based modifications (Haile et al., 2016). At the cellular level, GSK583 inhibited TNF-*α* and IL-8 secretion and the activation of the NOD-like receptor signaling pathway. In addition, GSK583 dose-dependently inhibited TNF-α and IL-6 production in intestinal mucosal tissue samples from patients with Crohn’s disease and ulcerative colitis, with an inhibitory effect comparable to that of the steroid prednisolone. Unfortunately, the off-target activity on the hERG ion channel and poor pharmacokinetic/pharmacodynamic (PK/PD) properties limited its further development (Haile et al., 2016). After multiple optimizations of GSK583, GSK2983559 (Figure 5H) was designed and validated through in vitro and *in vivo* experiments. Despite suboptimal solubility, it exhibited more favorable cross-species pharmacokinetics and demonstrated good efficacy in a mouse IBD model and UC/CD explants. It later became the first RIPK2 inhibitor to enter clinical trials, but the trial was terminated due to nonclinical toxicology results and reduced safety thresholds (Haile et al., 2020; Haile et al., 2019) (ClinicalTrials.gov identifier: NCT03358407).

Various ALK2 inhibitors have also been found to inhibit RIPK2 activity (Mohedas et al., 2013; Mohedas et al., 2014). Based on a 3,5-diphenyl-2-aminopyridine scaffold, CSLP37 (Figure 5I) was developed to inhibit RIPK2 kinase activity and effectively block NOD2 signaling, while showing over 20-fold selectivity for ALK2 (Suebsuwong et al., 2020). Using a pyrido[2,3-d]pyrimidin-7-one scaffold, researchers developed UH15-15 (Figure 5J) by optimizing interactions with the Ser25 residue and the αC helix region, leading to strong efficacy and selectivity in inhibiting RIPK2 kinase activity and blocking NOD2 signaling, with favorable in vivo pharmacokinetics (Nikhar et al., 2021).

BI 706039 (Figure 5K) is a RIPK2-specific inhibitor that blocks MDP-induced TNF-α production in human and murine cells, with good selectivity and pharmacokinetics. In the TRUC mouse IBD model, BI 706039 significantly reduced colonic inflammation and disease-associated lipocalin levels in a dose-dependent manner (Ermann et al., 2021).

### 5.2 Type II kinase inhibitors and their derivatives

Type II kinase inhibitors target the inactive DFG-out conformation of kinases, offering better specificity than type I inhibitors. Using a fluorescence-based thermal shift assay, several type II RIPK2 inhibitors were identified: ponatinib (Figure 5K), regorafenib (Figure 5L), and sorafenib (Figure 5M). Cellular experiments confirmed their ability to inhibit RIPK2 autophosphorylation, ubiquitination, and NOD2 signaling activation (Canning et al., 2015). These findings were later validated in Goncharov’s study (Goncharov et al., 2018). The crystal structure of the RIPK2-ponatinib complex revealed a large allosteric pocket, due to Ala73 on the RIPK2 αC helix, occupied by ponatinib’s trifluoromethyl group. This pocket offers space for the design of larger chemical groups, increasing the selectivity and potency of inhibitors. Targeting this unique pocket could enhance specificity for RIPK2 while avoiding off-target effects on kinases like RIPK1 and RIPK3, providing a foundation for developing more selective RIPK2 inhibitors (Canning et al., 2015).

Subsequent virtual screening and molecular docking identified RIPK2 inhibitor 1 (Figure 5N), which displayed a binding mode similar to ponatinib, blocking RIPK2 autophosphorylation and NF-κB signaling, and reducing lung and intestinal inflammation (Salla et al., 2018).

To further improve specificity, researchers modified regorafenib by introducing a carboxylic acid fragment targeting the non-conserved RIPK2 activation loop, producing CSR35 (Figure 5O). This strategy provided proof of concept for targeting the RIPK2 activation loop (Suebsuwong et al., 2018).

While initial studies emphasized RIPK2 kinase activity in NOD-like receptor signaling, recent research suggests that the kinase domain may function primarily as a scaffold (Hrdinka et al., 2018; Goncharov et al., 2018), challenging the theoretical basis of some current inhibitors. Focusing solely on kinase inhibition might overlook other potential drug targets. With advances in technology, exploring the structural changes of RIPK2 during NOD-like receptor signaling activation using various sequencing techniques may uncover new druggable targets, potentially opening new avenues for the development of RIPK2-targeted therapies.

## 6 Conclusion

RIPK2 is a key mediator in immune signaling, especially downstream of NOD-like receptor pathways. Its roles extend across immune responses, inflammation, cancer progression, and cardiovascular diseases. This review highlights RIPK2’s structural and functional significance, along with advancements in RIPK2 inhibitor development.

While initial focus has been on RIPK2’s kinase activity, recent evidence suggests its primary function may be as a scaffold for ubiquitination and signaling. This shift highlights the potential for targeting non-kinase domains, such as the CARD domain or allosteric sites, to develop more selective therapies.

Our proposed model emphasizes RIPK2’s dynamic interaction with NOD-like receptors, particularly its transient oligomerization during bacterial infection. This may help explain some of the current phenomena. Current kinase inhibitors, while promising, face challenges in selectivity due to the conserved ATP binding pocket. The discovery of RIPK2-specific allosteric pockets opens avenues for more selective drug design, but further research is needed to optimize these inhibitors. Moreover, RIPK2’s non-canonical roles, including its involvement in autophagy and immune evasion, suggest untapped therapeutic potential.

## 7 Future outlook

### 7.1 Identification of direct substrates

As a dual-specificity kinase, the discovery of RIPK2’s direct substrates remains incomplete. Uncovering these substrates will be crucial to fully understand its signaling mechanisms.

### 7.2 Transient interactions

Future studies should aim to clarify the transient and dynamic interactions between RIPK2 and NOD-like receptors under physiological conditions. This will uncover new regulatory mechanisms that are currently not well understood.

### 7.3 Non-kinase functions

Since RIPK2’s role extends beyond its kinase activity, it is essential to explore its non-canonical functions in adaptive immunity, tumorigenesis, and other cellular contexts, expanding the scope of potential therapeutic targets.

### 7.4 Selective drug design

More selective RIPK2 inhibitors are needed, especially targeting the ATP binding site or allosteric sites. This will improve specificity while minimizing off-target effects, providing new strategies for treating inflammatory diseases, cancer, and beyond.
